# Supplementary material for: Structure and dynamics of 2x(CENP-A/H4)2 octasome reveal a possible intermediate in centromeric chromatin
Source: Life Sci Alliance. 2025 Dec 15;9(3):e202503377. doi: 10.26508/lsa.202503377 (PMC12705856; doi:10.26508/lsa.202503377)
Supplement: Supplementary file 1 [file LSA-2025-03377_SdataFS1.pdf]

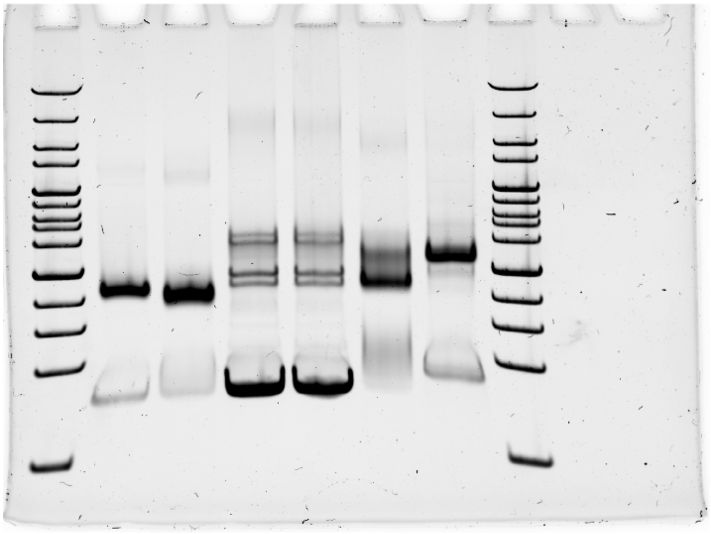

1- Uncropped gel figure S1A

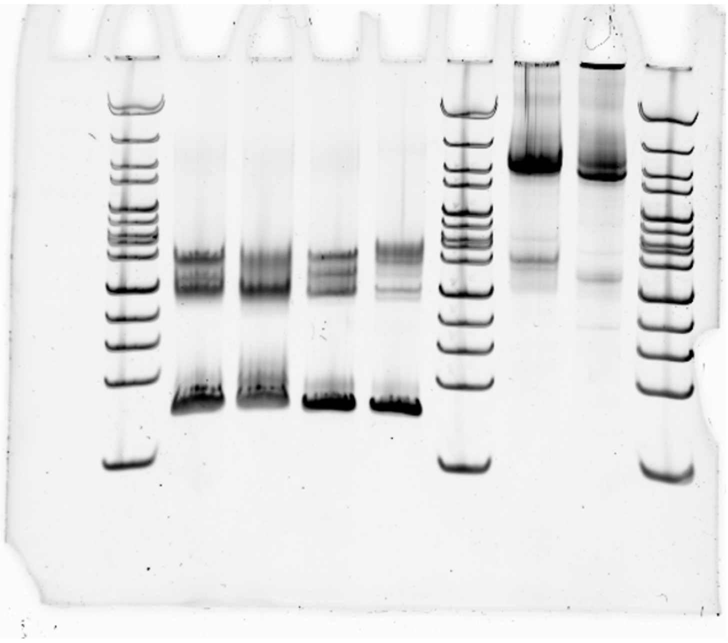

2- Uncropped gel figure S1B

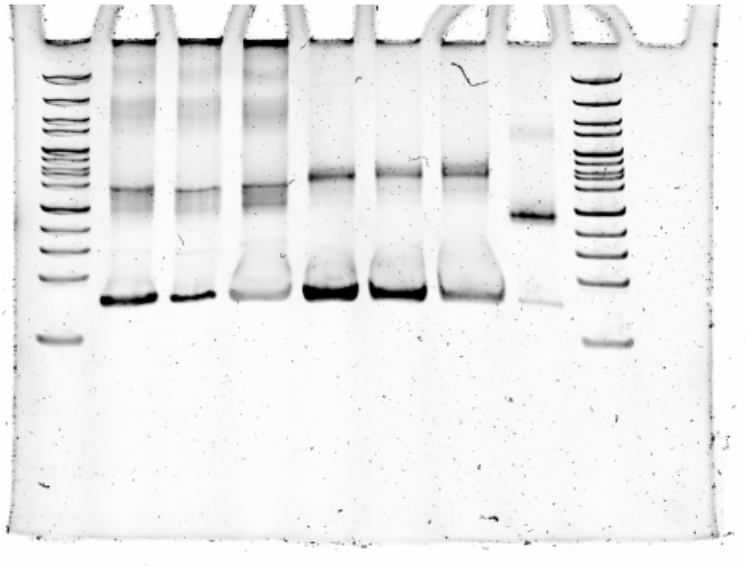

3- Uncropped gel figure S1C (top)

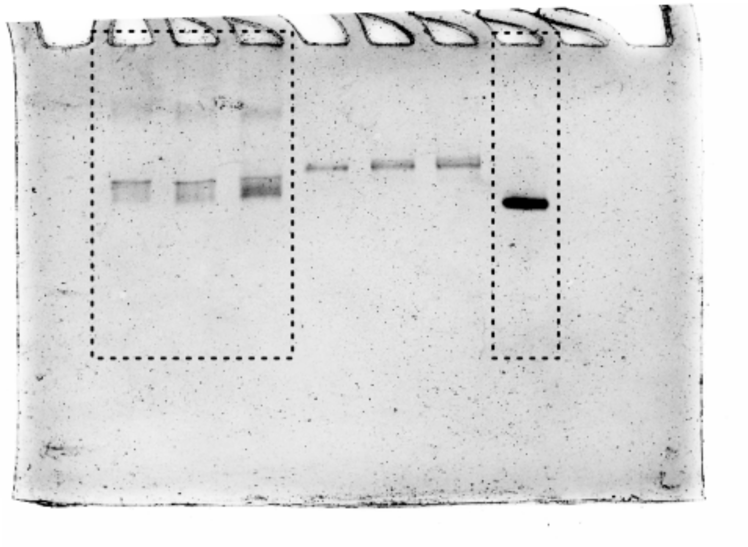

4- Uncropped gel figure S1C (bottom)

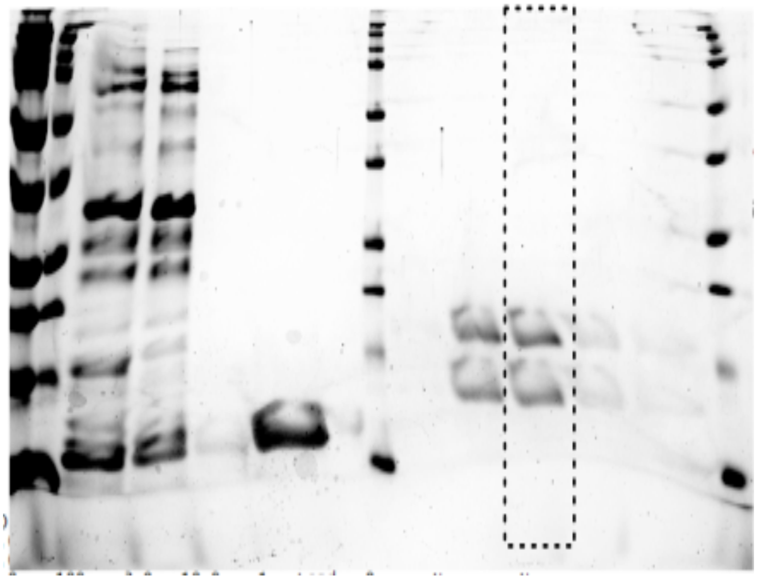

5- Uncropped gel figure S1D (CENP-A/H4)

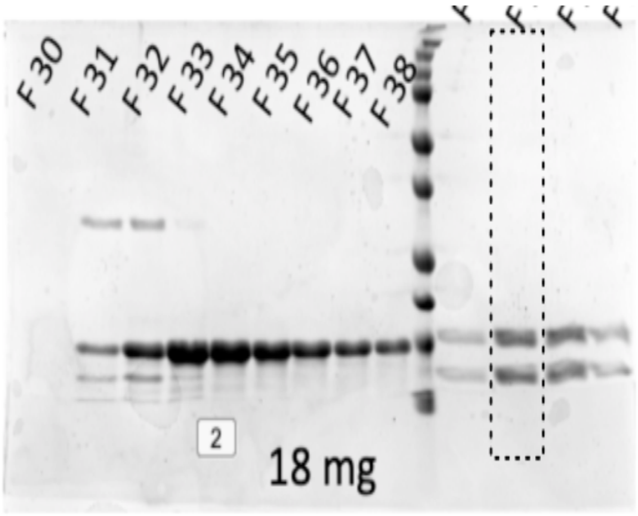

6- Uncropped gel figure S1D (H3/H4)

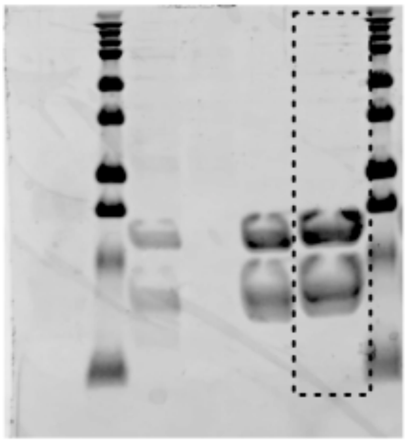

7- Uncropped gel figure S1D (CENP-A/H4 octasome)

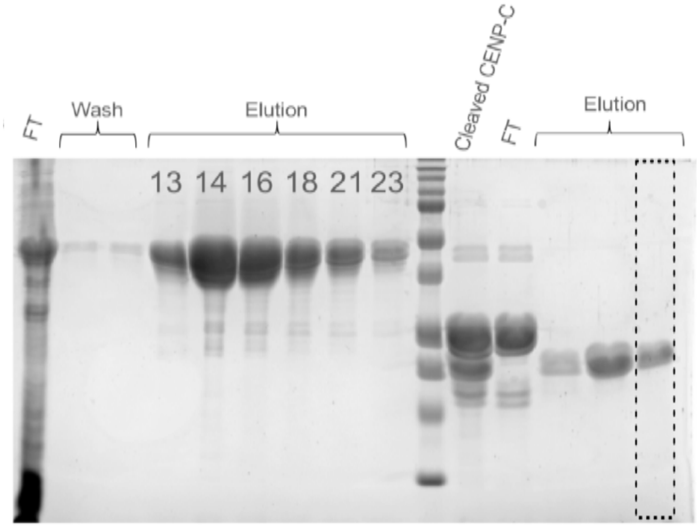

8- Uncropped gel figure S1D (CENP-C)

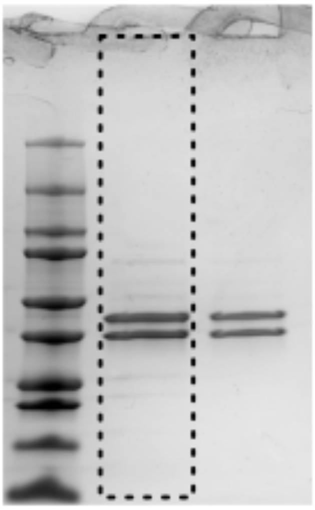

9- Uncropped gel figure S1D (CENP-N/L)

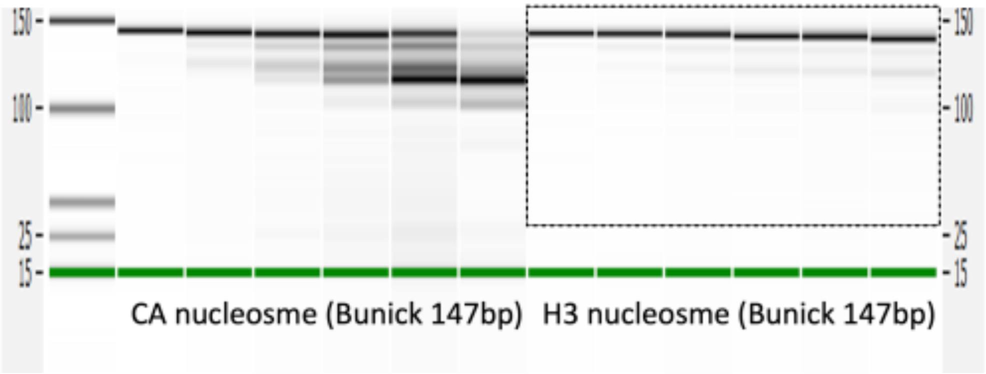

10- Uncropped gel figure S1E (CENP-A and H3 nucleosomes)

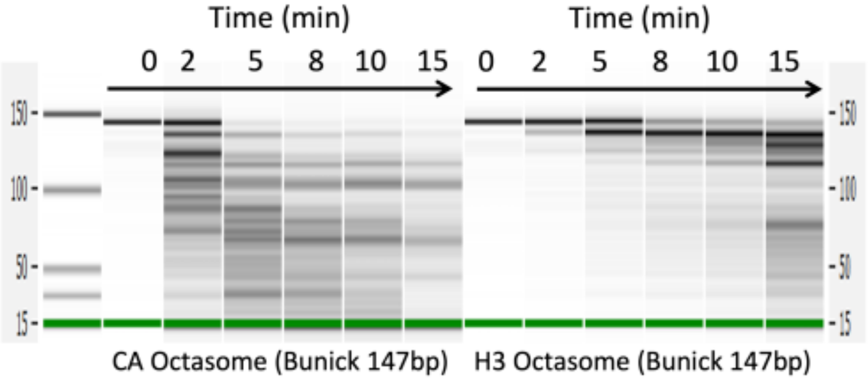

11- Uncropped gel figure S1E (CENP-A and H3 octasomes)

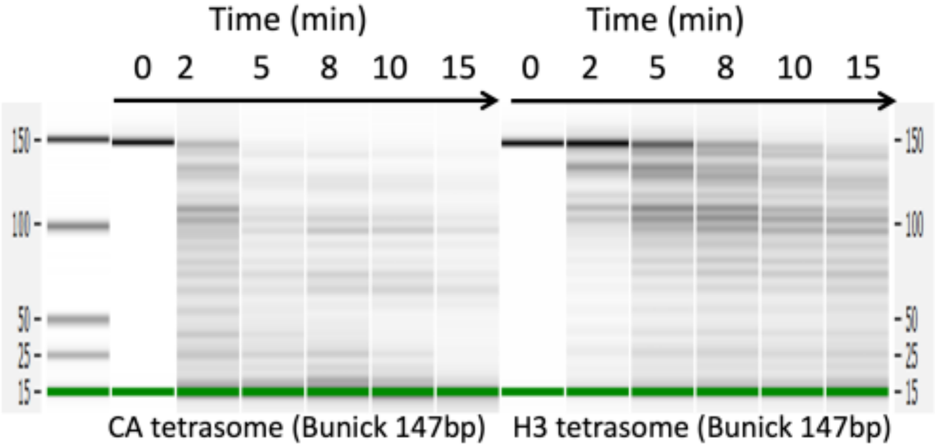

12- Uncropped gel figure S1E (CENP-A and H3 tetrasomes)
