# Supplementary material for: Structure and dynamics of 2x(CENP-A/H4)2 octasome reveal a possible intermediate in centromeric chromatin
Source: Life Sci Alliance. 2025 Dec 15;9(3):e202503377. doi: 10.26508/lsa.202503377 (PMC12705856; doi:10.26508/lsa.202503377)
Supplement: Supplementary file 2 [file LSA-2025-03377_TableS1.docx]

**Table S1. Cryo-EM data collection statistics for 2x(CENP-A/H4)_2_ and 2x(H3/H4)_2_ octasomes**

| **Data collection** |  |  |
| --- | --- | --- |
| Magnification | 165 000x |  |
| Voltage (kV) | 300 |  |
| Electron exposure (e–/Å^2^) | 57.5 |  |
| Defocus range in μm (steps) | -1.5 – -3.0 (0.3) |  |
| Pixel size (Å) | 0.82 | 0.82 |
| **2x(H3/H4)_2_** ^α-sat^ **octasome data processing** | **Open conformation**  EMD-51647  (Most unwrapped) | **Closed conformation**  EMD-51646  (Most wrapped) |
| Initial reference map | ab initio | ab initio |
| Symmetry imposed | C1 | C1 |
| Initial particle images (no.) | 158.3 K | 158.3 K |
| Final particle images (no.) | 64.7 K | 57.5 K |
| Box size (Å) | 360 | 360 |
| Pixel size (Å) | 0.82 | 0.82 |
| Map resolution (Å), FSC threshold | 3.75 | 3.82 |
| **2x(CENP-A/H4)_2_**^α-sat^ **octasome data processing** | **Closed conformation**  EMD-51645  PDB: 9GXA  (Most wrapped) |  |
| Initial reference map | ab initio |  |
| Symmetry imposed | C1 |  |
| Initial particle images (no.) | 119.3 K |  |
| Final particle images (no.) | 22.5 K |  |
| Box size (Å) | 360 |  |
| Pixel size (Å) | 0.82 |  |
| Map sharpening *B* factor (Å^2^) | -84 |  |
| Map resolution (Å), FSC threshold | 4.01 |  |
| **Refinement** |  |  |
| Initial model used | - |  |
| Model resolution (Å) FSC threshold | 4 |  |
| Model composition  Nonhydrogen atoms  Protein residues  Nucleotides  Ligands | 9226  575  224  0 |  |
| R.m.s. deviations  Bond lengths (Å)  Bond angles (°) | 0.004  0.652 |  |
| Validation  MolProbity score.  Clashscore  Poor rotamers (%) | 1.7  15.93  0 |  |
| Ramachandran plot  Favored (%)  Allowed (%)  Disallowed (%) | 98.39  1.61  0 |  |
