# Supplementary material for: Structure and dynamics of 2x(CENP-A/H4)2 octasome reveal a possible intermediate in centromeric chromatin
Source: Life Sci Alliance. 2025 Dec 15;9(3):e202503377. doi: 10.26508/lsa.202503377 (PMC12705856; doi:10.26508/lsa.202503377)
Supplement: Supplementary file 3 [file LSA-2025-03377_TableS2.docx]

**Table S2. Cryo-EM data collection statistics for (CENP-A/H4)_2_ poly-tetrasome.**

| **Data collection** |  |
| --- | --- |
| Magnification | 165 000x |
| Voltage (kV) | 300 |
| Electron exposure (e–/Å^2^) | 50 |
| Defocus range in μm (steps) | -0.8 – -2.4 (0.3) |
| Pixel size (Å) | 0.704 |
| **4x(CENP-A/H4)_2_** ^di-α-sat^ **tetra-tetrasome data processing** | EMD-51656 |
| Initial reference map | ab initio |
| Symmetry imposed | C1 |
| Initial particle images (no.) | 76.8 K |
| Final particle images (no.) | 27 K |
| Box size (Å) | 280 |
| Pixel size (Å) | 1.3074 |
| Map resolution (Å), FSC threshold | 7.01 |
