# Supplementary material for: Structure and dynamics of 2x(CENP-A/H4)2 octasome reveal a possible intermediate in centromeric chromatin
Source: Life Sci Alliance. 2025 Dec 15;9(3):e202503377. doi: 10.26508/lsa.202503377 (PMC12705856; doi:10.26508/lsa.202503377)
Supplement: Supplementary file 4 [file LSA-2025-03377_TableS3.docx]

**Table S3. Number of atoms composing the simulated systems, and total simulation time.**

|  | Protein/DNA | Water | Na^+^ | Cl^-^ | Total | Simulated time |
| --- | --- | --- | --- | --- | --- | --- |
| 3LZ0 | 24’751 | 757’812 | 864 | 723 | 784’150 | 900 ns |
| 6O1D | 25’862 | 756’285 | 865 | 724 | 783’736 | 900 ns |
| 2x(H3/H4)_2_ | 24’936 | 757’368 | 860 | 719 | 783’883 | 900 ns |
| 2x(CENP-A/H4)_2_ | 25’236 | 756’900 | 872 | 731 | 783’739 | 900 ns |
