# Supplementary material for: Structure and dynamics of 2x(CENP-A/H4)2 octasome reveal a possible intermediate in centromeric chromatin
Source: Life Sci Alliance. 2025 Dec 15;9(3):e202503377. doi: 10.26508/lsa.202503377 (PMC12705856; doi:10.26508/lsa.202503377)
Supplement: Supplementary file 6 [file LSA-2025-03377_SdataFS7.pdf]

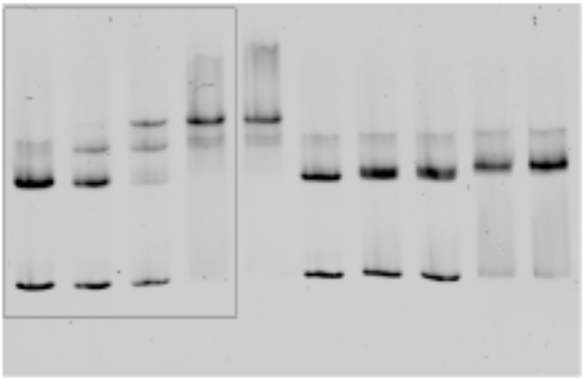

1- Uncropped gel figure S7A (CENP-C binding to CENP-A nucleosome)

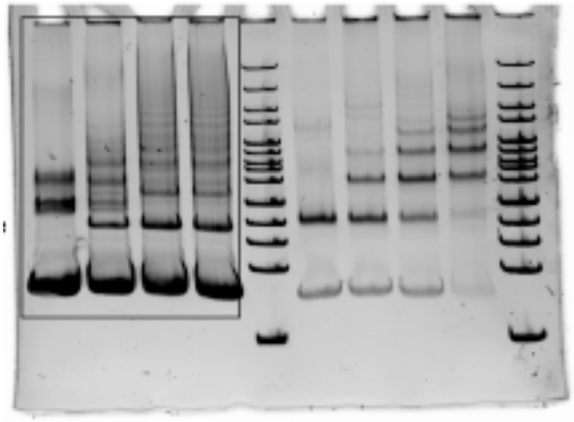

2- Uncropped gel figure S7B (CENP-N binding to CENP-A octasome)

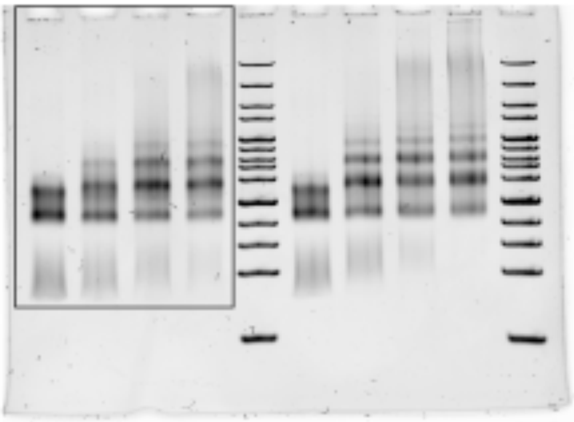

3- Uncropped gel figure S7B (CENP-N binding to H3 octasome)

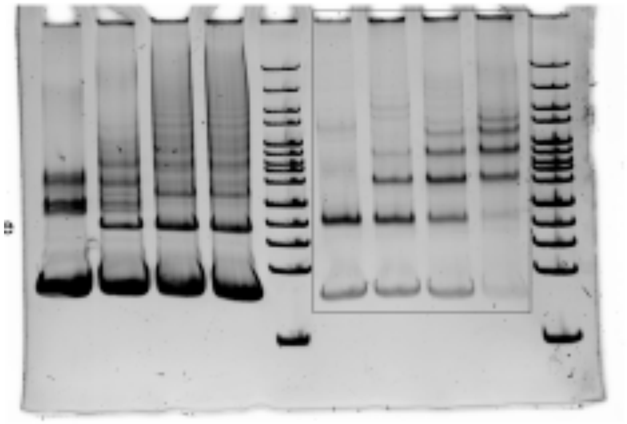

4- Uncropped gel figure S7B (CENP-N binding to CENP-A nucleosome)
