# Supplementary material for: Structure and dynamics of 2x(CENP-A/H4)2 octasome reveal a possible intermediate in centromeric chromatin
Source: Life Sci Alliance. 2025 Dec 15;9(3):e202503377. doi: 10.26508/lsa.202503377 (PMC12705856; doi:10.26508/lsa.202503377)
Supplement: Supplementary file 7 [file LSA-2025-03377_SdataFS10.pdf]

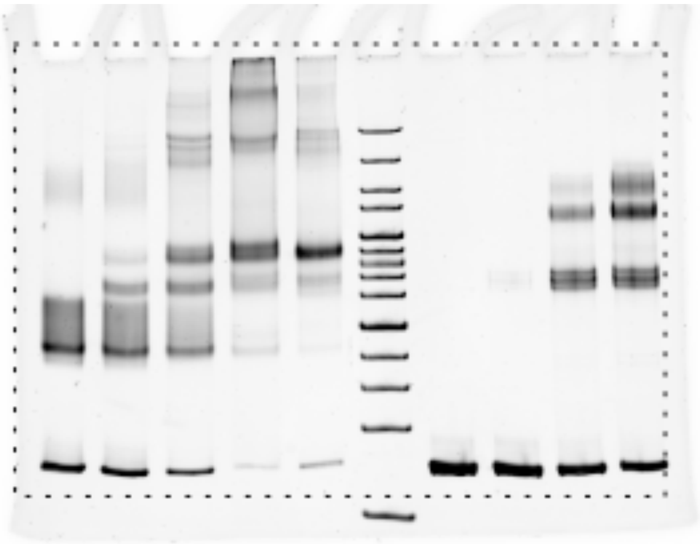

1- Uncropped gels figure S10 (Octasome:GFP-H2A/H2B titration)

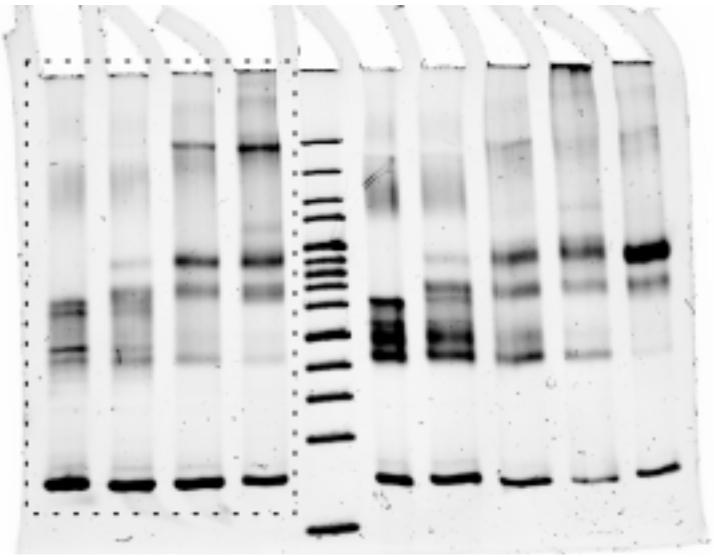

2- Uncropped gels figure S10 (Octasome:GFP-H2A/H2B titration)

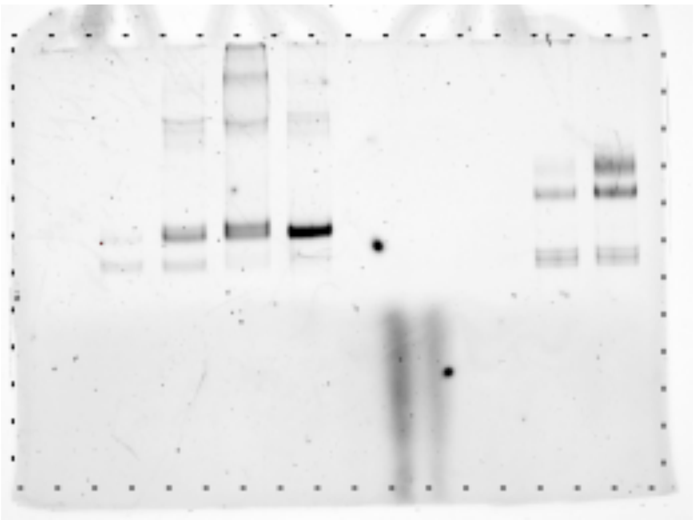
3- Uncropped gels figure S10 (Octasome:GFP-H2A/H2B titration)

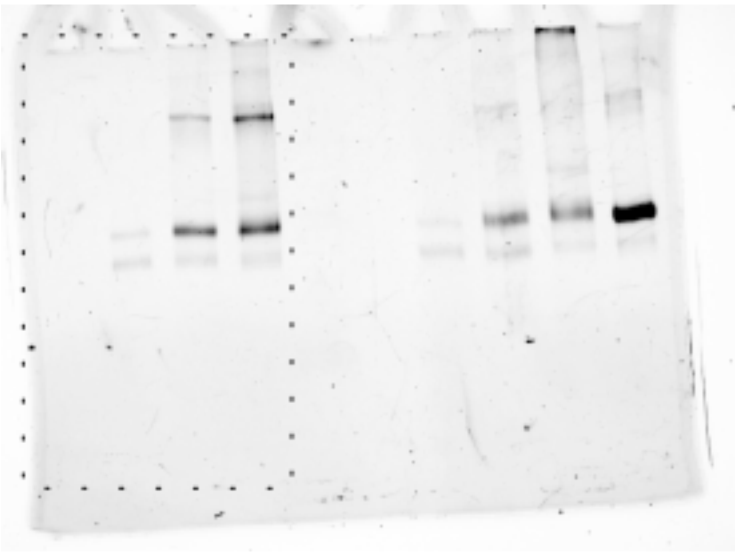
4- Uncropped gels figure S10 (Octasome:GFP-H2A/H2B titration)
