# Supplementary material for: Structure and dynamics of 2x(CENP-A/H4)2 octasome reveal a possible intermediate in centromeric chromatin
Source: Life Sci Alliance. 2025 Dec 15;9(3):e202503377. doi: 10.26508/lsa.202503377 (PMC12705856; doi:10.26508/lsa.202503377)
Supplement: Supplementary file 8 [file LSA-2025-03377_TableS4.docx]

**Table S4. Gyre data.**

|  | Gyre distance  Grye angle  (MD data) | Gyre distance  Gyre angle  (PDB) |
| --- | --- | --- |
| 3LZ0 | 21.1 ± 1.2 Å  14.7 ± 0.8 deg | 22.7 Å  15.7 deg |
| 6O1D | 21.8 ± 1.1 Å  15.3 ± 0.8 deg | 26.4 Å  17.9 deg |
| 2x(H3/H4)_2_ | 33.5 ± 0.8 Å  26.6 ± 0.6 deg | 36.8 Å  26.4 deg |
| 2x(CENP-A/H4)_2_ | 41.3 ± 1.0 Å  28.9 ± 0.9 deg | 40.2 Å  28.4 deg |
